# A case report of fatal familial insomnia with cerebrospinal fluid leukocytosis during the COVID-19 epidemic and review of the literature

**DOI:** 10.1080/19336896.2023.2298520

**Published:** 2024-01-16

**Authors:** Zheng Wang, Yueqi Huang, Shuqi Wang, Jiefang Chen, Gesang Meiduo, Man Jin, Xiaoying Zhang

**Affiliations:** Affiliated Mental Health Center & Hangzhou Seventh People’s Hospital, Zhejiang University School of Medicine, Hangzhou, China

**Keywords:** Clinical features, CSF analysis, fatal family insomnia, PET-CT, PRNP, Tau protein

## Abstract

Fatal familial insomnia (FFI) is a rare autosomal dominant genetic neurodegenerative disease. Generally, FFI patients will develop rapidly progressive dementia, sleep disturbance, autonomic dysfunction, and so on. Cerebrospinal fluid examination of FFI patients normally shows no obvious abnormalities. Here, we report a young male patient who was diagnosed with FFI during the COVID-19 epidemic. Clinical symptoms include psychobehavioral abnormality, cognitive decline, sleep disturbance, and autonomic dysfunction. No abnormalities were found in routine examinations after admission. However, the number of white blood cells in the cerebrospinal fluid increased. Though the patient was treated with anti-infection and immunotherapy, the symptoms were not relieved. A lumbar puncture was performed again, and it was found that the total Tau protein in the cerebrospinal fluid was elevated, and PET results showed that brain metabolism decreased. Finally, a genetic test was used to confirm the diagnosis of FFI. This case suggests that patients with FFI may also have elevated white blood cells in cerebrospinal fluid and timely detection of Tau protein in cerebrospinal fluid is helpful for early identification of FFI. And precise diagnosis relies on genetic testing.

## Introduction

FFI, a rare autosomal dominant disease, is associated with the D178N-129 M missense mutations of the prion protein gene (PRNP) on chromosome 20, and the main pathological feature is scrapie prion protein (PrP^Sc^) deposition in the brain [1,2]. The average onset age of FFI is about 51 years old, and the course of the disease ranges from 8 to 72 months [3]. Clinical symptoms of FFI patients are variable. Typically, FFI patients are accompanied with rapidly progressive dementia, sleep disturbance, autonomic dysfunction, and so on. Eventually, it will lead to death [4].

In this article, we report one case of a young male patient diagnosed with FFI who had been vaccinated against COVID-19 during the COVID-19 pandemic. This patient had no positive family history but with severe sleep disturbances, progressive cognitive decline, significant changes in autonomic nervous function, and psychobehavioral abnormality. Early detection of cerebrospinal fluid showed increased white blood cells. Multiple magnetic resonance imaging (MRI) and electroencephalography (EEG) examination showed no abnormalities, and the effects of anti-infection and immunotherapy treatment were poor. Finally, the patient was diagnosed with FFI by genetic testing.

## Case presentation

The patient, a 28-year-old courier, was admitted to the hospital in July 2021, because of ‘Memory impairment and psychotic behaviour disorder for more than one month’. The patient suffered from memory loss one month before admission without obvious incentives. He often made repeated mistakes at work and forgot whether he had eaten. There are often involuntary movements of limbs and frequent changes in his body position during sleep. Later, hallucinations and delusion gradually appeared. When his brother was not at home, he claimed to have seen him. He also said that his child was adopted, and he had another one. The patient had seen a doctor in another hospital before coming to our department. He was diagnosed with ‘anxiety disorder’ and was treated with ‘Olanzapine Tablets 2.5 mg/day’. After treatment, the patient’s sleep quality has slightly improved, but he often moves his hands and feet around during sleep and tears the quilt with his hands and teeth.

When admitted to the hospital, the patient’s body temperature was normal, his consciousness was clear, his facial expression was stolid, and his feeling of time orientation was not good. His short-term memory was impaired. He could not recall three things (ball, flag, tree) we told him five minutes ago. Both ankle reflexes were active and the right ankle clonus was positive. The patient’s father died due to a car accident in his forties. But his mother, sister, and daughter were all in good health. There was no similar medical history in his family. In February 2021, the patient had a history of vaccination against the novel coronavirus, and the patient’s wife reported that he had experienced erectile dysfunction two months before the disease onset.

**Figure 1.**
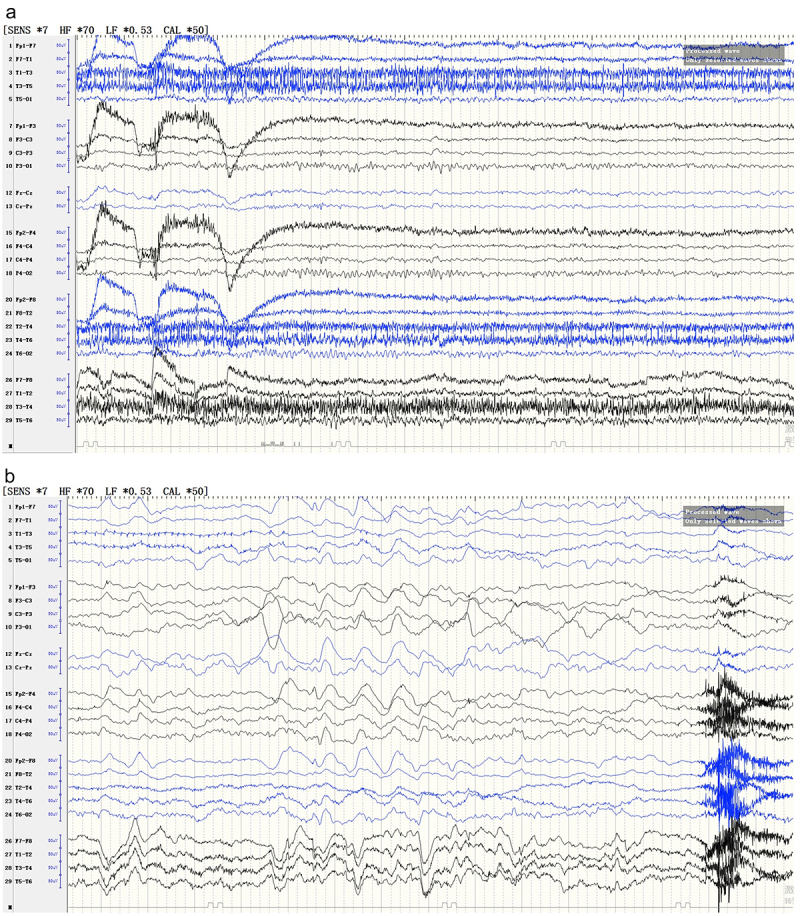
EEG of the patient.

**Figure 2.**
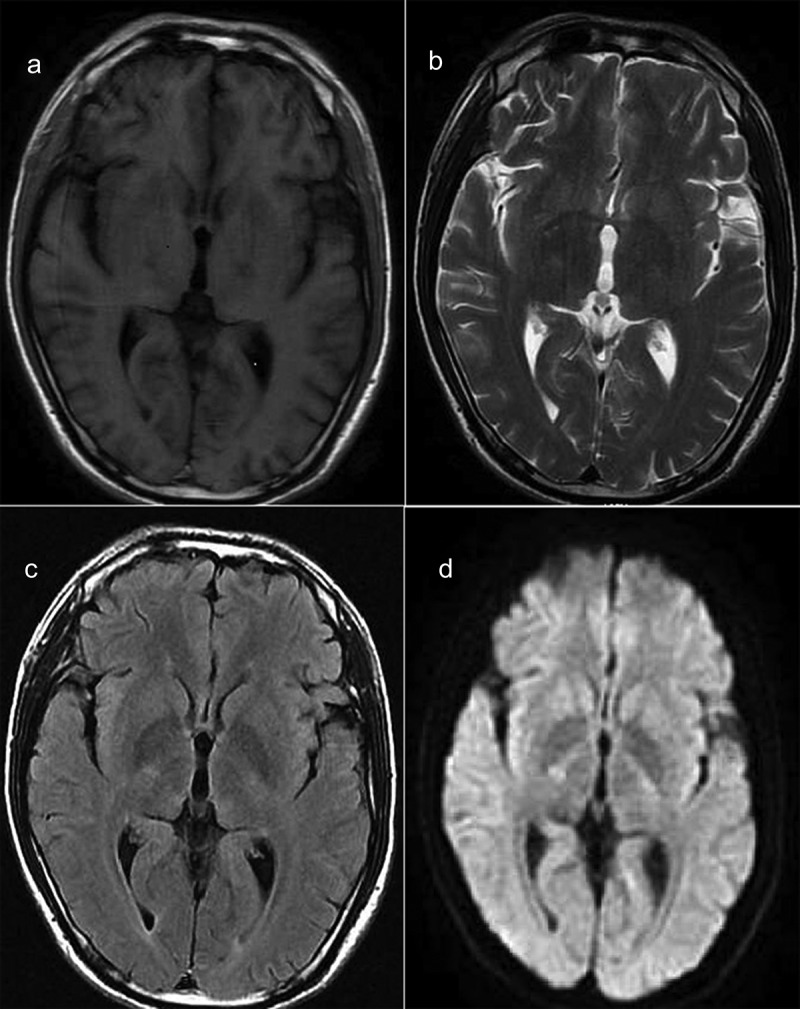
MRI of the patient.

The examination was completed immediately after the patient’s admission. The results showed that the amount of anti-thyroglobulin antibody (TG-Ab) (113.40IU/mL) and anti-thyroid peroxidase antibody (TPO) (60.97IU/mL) elevated slightly. No abnormalities were found in the tests of renal function, folic acid, vitamin B12, syphilis, HIV, and PCR of COVID-19, and no obvious abnormalities were found in the awake electroencephalogram(EEG) (Figure 1) and head MRI (Figure 2). The patient underwent lumbar puncture, and the cerebrospinal fluid pressure was 100mmH_2_O, the cerebrospinal fluid total protein was 0.39 g/l, and the amount of red blood cells was 1.68 × 10^9^/L, the amount of white blood cells was 36.60 × 10^6^/L↑ (After excluding blood contamination using the following formula: WBC in cerebrospinal fluid – WBC in blood*RBC in cerebrospinal fluid/RBC in blood, the white blood cell count is still high) (Table 1). However, when it comes to the detection of cerebrospinal fluid autoimmune encephalitis-related antibodies, serum paraneoplastic-related antibodies, and cerebrospinal fluid pathogen infection DNA and RNA, the results were all negative.

(a) EEG at the first hospitalization. The EEG showed normal background activity. A 10-HZ alpha rhythm can be seen during the closed eyes state. No abnormal slow waves were observed. (b) Re-examination of EEG at the second hospitalization. EEG showed diffuse and paroxysmal slow activity in bilateral brain regions.

MRI did not show significant abnormal hyperintensity in the cingulate, caudate, putamen, and cortical gyrus.

(a) T1 sequence (b) T2 sequence (c) T2Flair sequence (d) Diffusion-Weighted Imaging(DWI) sequence.

**Table 1.** Data details of cerebrospinal fluid of three lumbar puncture.

	RBC	WBC	Lymphocytes percentage	Protein
First lumbar puncture	1.68 × 10^9^/L	36.60 × 10^6^/L	12.3%	0.39 g/L
Second Lumbar Puncture	0.09 × 10^9^/L	2.70 × 10^6^/L		0.46 g/L
Third Lumbar Puncture	0.04 × 10^9^/L	0.30 × 10^6^/L		0.27 g/L

After excluding blood contamination using the following formula: WBC in cerebrospinal fluid – WBC in blood*RBC in cerebrospinal fluid/RBC in blood, the white blood cell count of first lumbar puncture is still high.

The patient’s clinical symptoms were subacute onset mental disorders and cognitive decline, with a high number of white blood cells in cerebrospinal fluid and no similar family disease history. These results suggested the possibility of viral encephalitis. Antibody-negative autoimmune encephalitis, metabolic disease, toxic encephalopathy, genetic disease, and neurodegenerative disease were taken into consideration as differential diagnosis. Regarding treatment, ‘Acyclovir injection 0.5 g q8h’ was administered intravenously. After 5 days of treatment, the patient’s symptoms were not relieved significantly. Combined with the increase in the patient’s thyroid-related antibodies, and the history of COVID-19 vaccination before the disease onset, self-immune-related encephalitis should be considered. ‘Methylprednisolone intravenous drip (160 mg/day, 5 days in total, then gradually reduce it) and human blood immunoglobulin intravenous drip (0.4 g/kg.d, 5 days in total)’ were given. And Acyclovir treatment continued for 2 weeks. Afterwards, a lumbar puncture was performed again, and the cerebrospinal fluid test showed the amount of white blood cell reduced to the normal range(2.70 × 10^6^/L) (Table 1). However, the patient’s symptoms became progressively worse. Memory decline and abnormal sleep behaviour became more obvious, and the speech act was confusing. The patient suffered from fragmentary visual and auditory hallucinations, accompanied by autonomic nervous system symptoms such as rapid heart rate and excessive sweating. Due to the poor treatment effect, the patient’s family members requested the discharge.

**Figure 3.**
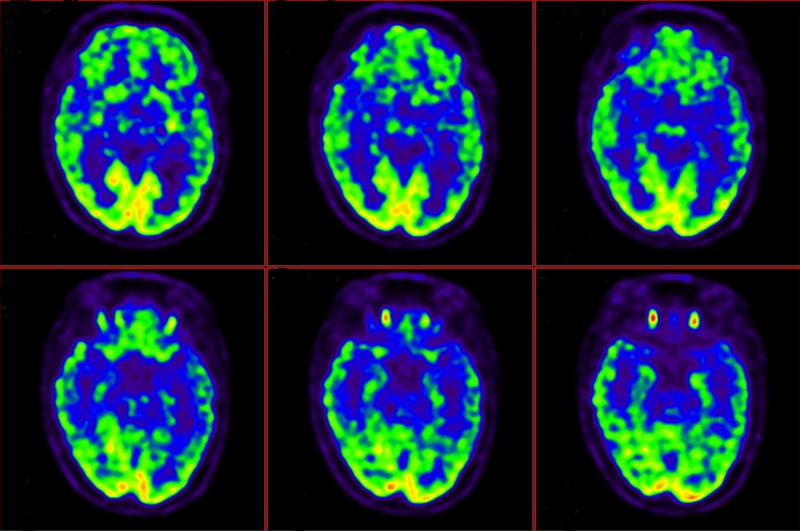
PET-CT of patient’s brain.

**Figure 4.**
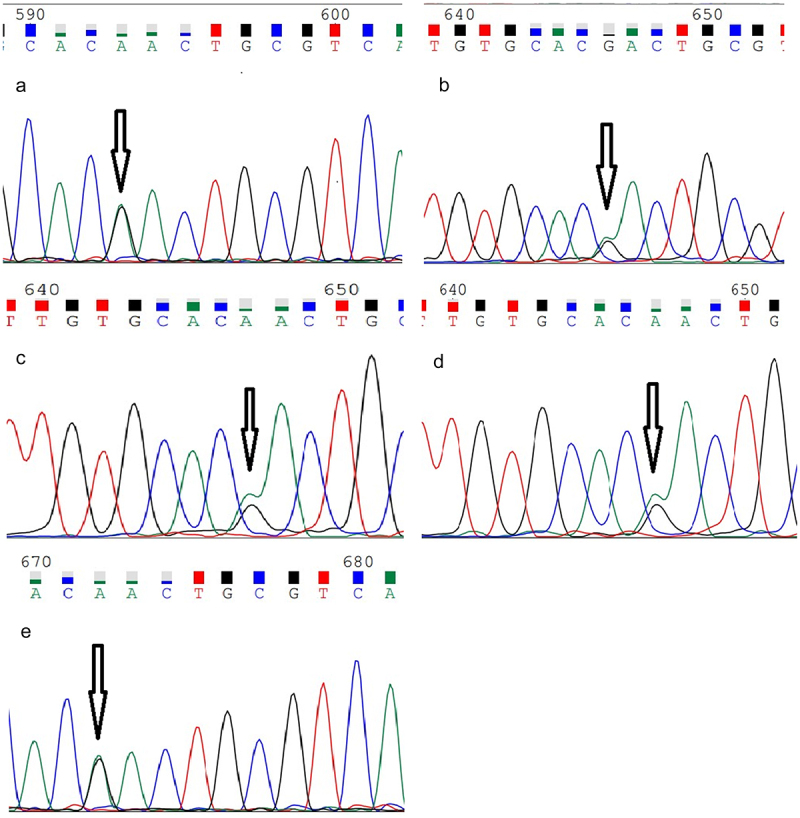
Results of prion protein gene detection (PRNP) in patients and family members.

In November 2021, the patient was admitted to our hospital again. The patient’s symptoms were worse than before. He showed a slurred speech, unsteady walking, wheezing, abnormal throat noise during sleep, and repeated getting up and groping. He could not recognize his family members, was unable to eat by himself, had a heavy sweat and a rapid heart rate. After admission, a re-examination of thyroid-related antibodies showed no abnormality. A lumbar puncture was performed again, and the cell number and protein amount in the cerebrospinal fluid were normal (Table 1). The results of theTau protein test in the cerebrospinal fluid indicated that phosphorylated Tau protein 181 (P-Tau) was 17.25 pg/ml, total Tau (T-Tau) was 453.48 pg/ml, and the T/P ratio was 26.29. Obviously, the Tau protein and T/P ratio were significantly elevated. EEG re-examination revealed diffuse and paroxysmal slow activity in bilateral brain regions without obvious spikes (Figure 1). PET-CT showed that the glucose metabolism in the cerebral cortex was reduced, especially in the frontal lobe (Figure 3). Reviewing the medical history, we found the patient’s symptoms continued to worsen after antiviral, immune regulation, and psychotropic drug treatment. Viral encephalitis, autoimmune encephalitis, and primary mental illness were basically ruled out. No suspicious metabolic aetiology was found in relevant blood tests. No history of poison exposure was found in the history, and the possibility of genetic disease was considerably high. To further clarify the cause, genetic testing was performed after obtaining the agreement of the family members. The result showed that the patient had a D178N-129 M missense mutation in the PRNP gene, which was consistent with FFI (Figure 4). Combined with the patient’s rapidly progressive cognitive impairment, abnormal sleep behaviour, and autonomic dysfunction, the patient was finally diagnosed with FFI.

PET-CT results suggested that the glucose metabolism in the cerebral cortex was unevenly distributed, and the metabolism in the frontal lobe was most obviously reduced.

(a) The peak map of the patient’s PRNP sequencing. There is a GA mutation (arrow) at position 593, that is, the D178N mutation. B-E indicate that the patient’s mother (b), sister (c), daughter (d), and nephew (e) have GA heterozygous mutations, respectively.

**Figure 5.**
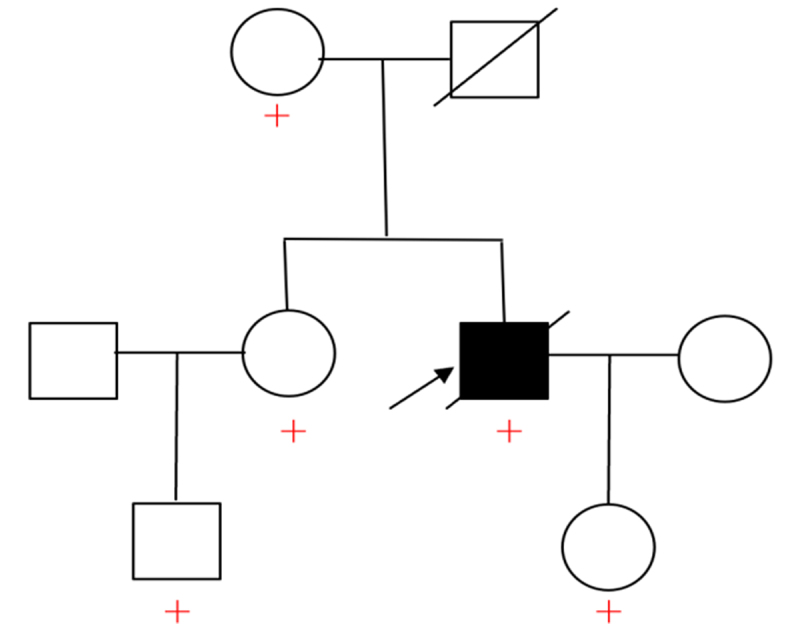
Family tree diagram.

Further genetic testing was carried out on the immediate family members of the patient, and it was found that the patient’s mother, sister, daughter, and nephew all had the same PRNP gene mutation (Figure 4). And the family tree diagram of the patient was made (Figure 5). Still, no relevant clinical symptoms have appeared so far among them. Unfortunately, the patient died of respiratory failure due to aspiration pneumonia only one month after the diagnosis.

The arrow points to the patient, + represents the presence of PRNP mutation gene, and the father of the patient died unexpectedly at an early age.

Although the diagnosis of FFI has been confirmed by genetic testing, we still hoped to complete the histopathological, immunohistochemical (IHC), or Western blot (WB) testing of prion aggregates to obtain a more definitive diagnosis. Unfortunately, the patient’s family refused the autopsy.

## Discussion

FFI is a rare autosomal dominant genetic disease. More than 100 cases have been reported worldwide so far since the first case of FFI was reported in detail in the New England Journal of Medicine in 1986 [5]. FFI has an acute or subacute onset and rapid progression.The main clinical symptoms are abnormal sleep-wake cycle, neuropsychiatric symptoms (including rapidly progressive dementia, ataxia, etc.), and progressive autonomic dysfunction (such as hypertension and rapid heart rate). The age of onset of the disease ranges from 17 to 76 years, and the course of the disease ranges from 2 to 48 months. Most of them have a clear family disease history [4].

The reported patient in this case had a relative earlier onset age, and there was no similar medical history in his family before his onset. And the number of white blood cells in the cerebrospinal fluid increased significantly in the early stage of the disease, reaching 36.60 × 10^6^/L, so it was diagnosed as ‘intracranial infection’ in the early stage. However, after anti-infection treatment, the patient’s symptoms still worsened, and the number of cells in the cerebrospinal fluid dropped to normal after a lumbar puncture. There are few reports on the changes of white blood cell count in the cerebrospinal fluid of patients with FFI. In several literatures, the number of white blood cells in the cerebrospinal fluid of FFI patients is mostly described in the normal range [6–8], but a study on the composition of cerebrospinal fluid in patients with prion disease showed that some Creutzfeldt-Jakob disease (CJD) patients had slightly elevated number of white blood cell count in the cerebrospinal fluid [9]. Cerebrospinal fluid leukocyte counts were elevated in 5 of 26 patients with hereditary CJD and 3 of 298 patients with sporadic CJD included in the study. The CSF white blood cell counts of these patients were all slightly elevated, but only one patient had a cerebrospinal fluid white blood cell count greater than 20/ul. However, none of the patients included in this study showed a simultaneous abnormal increase in WBCs, total protein, and oligoclonal IgG in cerebrospinal fluid. As in our case, there was an increase in WBCs in the cerebrospinal fluid but the total protein content was normal. It suggests that the increase of white blood cells in the cerebrospinal fluid of patients with prion disease is not due to the classic inflammatory response, but to other reasons which are still unclear [9]. Although the increase of white blood cells in the cerebrospinal fluid of patients with prion disease will bring difficulties to doctors when it comes to the diagnosis, we believe that for patients with cerebrospinal fluid leukocytosis, early ant-infection treatment should still be given in cases where the diagnosis is not yet clear, to avoid adverse consequences caused by delay in treatment.

In addition, the patient’s onset was during the COVID-19 pandemic, and he had a history of vaccination before the onset of the disease. Early test results showed that autoantibodies and thyroid-related antibodies increased, so it was considered that there might be autoimmune-related encephalitis in the early stage of the disease. Cases of elevated thyroid-associated antibodies in the blood of patients with prion disease have been reported before. Cossu and Jang both reported a CJD case with increased thyroid antibody (anti-thyroid peroxidase antibody (TPO-Ab) and anti-thyroglobulin antibody (Tg-Ab)) in 2003 and 2014, respectively. The two patients were both diagnosed as Hashimoto’s encephalitis at the early stage, but the disease continued to worsen after corticosteroid treatment [10,11]. In our case, clinical symptoms continued to worsen after corticosteroids and immunoglobulin therapy. However, in our opinion, early corticosteroid therapy is still necessary for patients with suspected autoimmune-associated encephalitis, because the response to corticosteroid therapy may be the only valid diagnosis method in the current stage before brain biopsy.

In addition to the early leukocyte elevation in the cerebrospinal fluid of this case, the Tau protein content was also abnormally elevated. Sanchez et al.‘s study showed that a T-Tau greater than 1300 pg/ml has some value for diagnosing sporadic Creutzfeldt-Jakob disease (sCJD) [12]. Other researchers have investigated the diagnostic significance of the ratio of total tau to phosphorylated tau [13–16]. An elevated ratio of total tau to phosphorylated tau levels has a specificity of 94–97% with a sensitivity ranging from 75–94% for CJD [17].

However, Anna’s study showed that the level of Tau protein in the cerebrospinal fluid of patients with genetic Creutzfeldt-Jakob disease(gCJD) also increased obviously, but not on patients with FFI [18].

Recently, a study on the critical value of genetic prion diseases (gPrD) cerebrospinal fluid markers showed that although the amount of Tau protein in the cerebrospinal fluid of patients with FFI is lower than that of sCJD, its elevated level still has diagnostic significance. When the T-Tau content of cerebrospinal fluid of a patient exceeds 284 pg/ml, the sensitivity and specificity of FFI diagnosis can reach 78% and 80%, respectively [19]. The cerebrospinal fluid test of this patient showed a P-Tau value of 17.25 pg/ml, a T-Tau value of 453.48 pg/ml, and the T/P ratio is 26.29. The T-Tau value is greater than 284 pg/ml. At the same time, a study conducted by Chen et al. on the 14-3-3 protein and T-Tau protein in the cerebrospinal fluid of Chinese patients with gPrD showed that 41.47% of FFI patients had Tau protein less than 2000 pg/ml, and T-Tau content in CSF of FFI patients with myoclonus is relatively high [20]. The patient in our case did not show myoclonus throughout the course of the disease, and the T-Tau content in the cerebrospinal fluid was relatively low, which was consistent with the findings of Chen et al.

Although Tau is valuable in diagnosis, it is not a specific biomarker for prion disease. Real-time quaking-induced conversion (RT-QuIC) is generally considered to be a specific method for the diagnosis of prion disease, which does not require brain tissue. It exploits the autocatalytic template directed protein misfolding nature of prions to propagate and aggregate when exposed to recombinant prion protein, and its output is detected, and measured, in real-time, by the fluorescence properties of protein aggregates [21]. The sensitivity of RT-QuIC ranges from 90.3 to 97.2%, with a specificity of 98.5–100% across all prion diseases [17]. In sCJD, sensitivity and specificity have been reported as high as 97 and 100%, respectively [22]. While RT-QuIC has decreased sensitivity in certain genetic and atypical sporadic prion disease, including FFI, GSS, sFI, VPSPr, and the VV1 and MM2 subtypes of sCJD [23]. Unfortunately, we did not consider prion disease as a differential diagnosis for the patient initially. After several ineffective treatments, we suspected that the patient had a genetic disorder and performed genetic testing. The D178N-129 M missense mutation in the PRNP gene was identified, and the patient was diagnosed with FFI. Therefore, we did not perform RT-QuIC testing for this patient.

In addition to RT-QuIC, Brain MRI also plays a fundamental role in the diagnosis of prion disease and in ruling out other diseases. MRI criteria of prion disease consists of hyperintensity in cingulate, caudate nucleus and putamen, and more than one neocortical gyrus or in more than three cortical gyri in either DWI or FLAIR [24,25]. In Forner’s research, MRI was found to have a sensitivity of 98% and specificity of 93% in sCJD [26]. But to our knowledge, FFI case rarely show typical neuroimaging abnormalities of CJD [27]. A Study Focusing on Quantitative Magnetic Resonance showed that patients with FI(fatal insomnia) presented significant atrophy in thalamus and cerebellum [28]. But this has no specificity in diagnosis.

The PET-CT examination of this patient was completed, and it was found that the brain metabolism was generally low, and the frontal lobe metabolism was significantly reduced. PET studies on FFI patients suggest that in the early stage of FFI, decreased metabolism in the thalamus is common, which may also be the reason why FFI patients often present with sleep disturbance and autonomic dysfunction in the early stage of the disease [29,30]. Cortelli et al. performed PET tests on the same FFI patient at different stages of the disease, and found that with the progression of the disease, the number of brain regions with reduced metabolism gradually increased, and the cingulate gyrus, limbic system, and other cerebral cortex could be involved, and the degree of reduced metabolism gradually increased. In the cortex, the areas most prone to hypometabolism and areas with the most severe reduction were the lateral frontal cortex and the frontal basal cortex (6 out of 7 FFI patients had hypometabolism in the frontal lobe), and the scope of reduced metabolism is more consistent with the distribution of PrPSc in brain tissue found in autopsy [30]. The patient’s course of disease had reached 6 months when the PET-CT examination was performed. Combined with the PET-CT examination results, it was suggested that the lesion had involved the cerebral cortex, mainly involving the frontal lobe. The prefrontal cortex is involved in advanced cognitive functions such as working memory, distraction and shifting of attention [31–33], which may be the reason for the patient’s cognitive dysfunction and some misconceptions.

Finally, it should be noted that, the patient’s mother, sister, daughter, and nephew all had the same PRNP gene mutation as the patient. But so far, only the patient himself has shown clinical symptoms of FFI. According to recent studies, D178N variants have evidence of high penetrance, conveying lifetime risk of > 90% [34,35]. But age of onset ranges from 12 to 89 years old for D178N variants [35]. And an individual’s age of onset does not appear to be predicted by their affected parent’s age of onset, including that there is a lack of systematic trend towards earlier onset in subsequent generations [35,36]. Therefore, despite good health by now, the family members’ risk of onset is still high in the future, and require long-term attention.

## Conclusion

During the COVID-19 epidemic, it is more challenging to find the aetiology of rapidly progressive dementia, especially in patients with elevated white blood cells in the cerebrospinal fluid. In addition to common causes, the possibility of prion disease cannot be ignored, especially when anti-infection therapy and immunotherapy treatment are useless. In the absence of a positive family history, RT-QuIC testing is of great guidance for the early identification of the disease, cerebrospinal fluid Tau protein testing is helpful for early identification, and genetic testing remains the gold standard for the disease. PET-CT examination is not specific to prion disease, but hypometabolism on PET should make clinicians consider neurodegenerative conditions, including prion disease, in the differential. Of course, for patients with rapidly progressive dementia, it is still necessary to actively search for a curable cause and give corresponding treatment when the diagnosis is not yet clear, to avoid the poor prognosis caused by treatment delay.
